# Changes in the Elemental and Metabolite Profile of Wheat Phloem Sap during Grain Filling Indicate a Dynamic between Plant Maturity and Time of Day

**DOI:** 10.3390/metabo8030053

**Published:** 2018-09-19

**Authors:** Lachlan J. Palmer, James C. R. Stangoulis

**Affiliations:** Biological Sciences, College of Science and Engineering, Flinders University, Bedford Park 5042, Australia; james.stangoulis@flinders.edu.au

**Keywords:** phloem sap, diurnal, metabolite profile, wheat, *Triticum aestivum*, micronutrients

## Abstract

The long distance transport of Fe and Zn in the phloem sap of wheat (*Triticum aestivum* L.) is the key route for seed supply, due to wheat having a xylem discontinuity. To date, our knowledge is limited on Fe and Zn homeostasis in the phloem sap during the reproductive and grain filling stages. With the use of aphid stylectomy to collect samples of phloem sap, we explored maturity and morning versus afternoon (within-day) changes in nutrient and metabolite profiles. Phloem exudate was collected from a wheat breeding line, SAMNYT16, at three times during the grain filling period and at both midday and mid-afternoon. There were significant changes in the concentration of Mg, K, Fe and Zn during the course of grain loading and there were also significant within-day differences for Fe and K concentrations in the phloem exudate during the early phases of grain development. We found that, for K and Fe, there was an increase of 1.1- and 1.4-fold, respectively, for samples taken prior to midday to those from mid-afternoon. There was also a significant decrease in K, Fe and Zn phloem sap concentration of 1.5-, 1.4- and 1.1-fold, respectively, from the start of peak grain loading to the end of grain loading. Of the 79 metabolites detected within samples of phloem exudate, 43 had significant maturity differences and 38 had significant within-day variability. Glutamine was found to increase by 3.3–5.9-fold from midday to mid-afternoon and citric acid was found to decrease by 1.6-fold from the start of grain loading to the end of grain loading. These two metabolites are of interest as they can complex metal ions and may play a role in long distance transport of metal ions. The work presented here gives further insight into the complex composition of the phloem sap and variability that can occur during the day and also with increasing maturity.

## 1. Introduction

Within a plant, the long distance transport pathways of the xylem and phloem are the major routes for nutrient movement from vegetative tissues to developing seeds [1]. An example of the importance of nutrient transport has been demonstrated with work done overexpressing ferritin in the endosperm of rice where grain Fe increased at the expense of leaf tissues [2]. In wheat and oats, the phloem plays a critical role as there is an interruption in the xylem at the base of each floret [3]. This discontinuity in the xylem means that macro- and micronutrients must be transported into the phloem before being available for unloading into the grain. Due to the reactive nature of Fe and Zn ions, these minerals are complexed during transport within the phloem [4] and modeling based on phloem exudate composition has theorized that Fe, Zn and other essential minerals are complexed with a variety of metabolites [5] and, of these, nicotianamine (NA), 2′-deoxymugineic acid (DMA) and cysteine are proposed to play a major role. In rice, NA has been found to complex Zn in the phloem sap [6]. For most cereals, phytosiderophores, which include NA and DMA, play an important role in uptake of Fe and Zn from the soil [7] and phytosiderophore secretion has also be shown to have diurnal regulation in wheat [8,9], rice [10,11] and barley [12]. While NA and DMA play a major role in chelating Fe and Zn, their concentration in phloem exudate does not necessarily indicate that they control the translocation of these metals in the phloem, as metals are translocated simply by mass flow and as sucrose is the predominant sugar in cereal phloem sap, its concentration plays a critical role in maintaining pressure gradients between source and sink and hence flow rates within the sieve elements.

In Arabidopsis, the homeostasis of Cu [13] and Fe [14] have been linked to circadian clock genes and, in the case of Fe, it has been shown that there is a link between the expression of Arabidopsis Ferritin 1 (AtFer1) and Time for Coffee (TIC) [14]. AtFer1 codes for a 28 kDa ferritin protein which in Arabidopsis has the highest expression under Fe excess [15], whilst TIC encodes a nuclear located regulator of the circadian clock responsible for maintaining amplitude and timing of the Arabidopsis circadian clock [16,17]. Ferritin production was found to be repressed by the expression of TIC under low Fe supply and in the presence of light and diurnal cycles [14]. Furthermore, links have been made between Fe and the regulation of circadian rhythms [18] and Fe homeostasis and light [19], with the Fe supply affecting the length of the circadian period [18] and a co-regulation between light and Fe acclimation responses [19]. Interactions between ferritin genes and light have also been observed in rice, with the presence of light stimulating the expression and production of ferritin in rice seedlings [20]. Ferritin is an important Fe storage protein with an important role in protecting against oxidative stress [15]. The interaction of diurnal patterns and Fe deficiency response within the roots of rice has also been explored [11] and the critical Fe complexing compound 2′-deoxymugineic acid (DMA) was found to follow a diurnal pattern in gene expression and production within the roots [11]. The role of the circadian rhythm in homeostasis of essential elements is of great interest. An important part of mineral translocation is the efficient transport of target micronutrients (i.e., Fe and Zn) from source to the sink (i.e., from soil, through to the roots, stems and leaves, and then to the seed) and therefore the long-distance vascular pathways play a significant role in delivering nutrients to seeds [21]. The effects of diurnal patterns observed in Fe homeostasis in various tissues of photosynthetic organisms [19] and more specifically the interaction between phytosiderophores and diurnal patterns in the roots of rice [11] point to the potential for changes in the phloem exudate nutrient concentration with the time of day.

These circadian rhythms also take place within the context of plant and grain development. In the case of wheat, it has been found that Zn loading into the grain of some genotypes peaks at between 10 and 14 days after anthesis [22,23]. The availability of Zn during this period is critical for the loading of Zn into the grain [22].

The connection between diurnal and circadian rhythm, plant development, nutrient flux and long distance transport is crucial when exploring the mechanisms of micronutrient unloading into the grain. These relationships are also important to ensure that variability caused by diurnal and circadian rhythms are considered when sampling exudate from the phloem. Very little is known about the direct effect of time of day on the composition of phloem exudate and this is mainly due to difficulties in the methods used for collecting phloem exudate samples and the small amounts collected [24]. Recent advances in analytical equipment have enabled the measurement of metabolites, elements and amino acids in volumes small enough to enable the examination of within-day changes directly within the phloem exudate [25,26,27]. In work examining the change in amino acid concentration within the phloem exudate from mid-day to late afternoon, the concentrations of 14 individual amino acids were reported in phloem exudate [27]. The concentrations for amino acids that could not be separated were also reported; these were histidine and valine (His/Val) and leucine and isoleucine (Leu/Ile) [27]. Of the amino acids measured, six individual amino acids and both combined amino acids were found to increase in concentration as the day progressed [27]. The concentrations of four elements [26] and 79 metabolites [25] have been shown to change in the phloem exudate of wheat during grain development. The work presented here aimed to determine if there is variability in phloem exudate concentration of Mg, K, Fe and Zn when sampled at different times of day and if this variability changes at different times of the grain loading period. We explored the variability in the phloem exudate metabolite profile with time of day at different maturities during grain loading.

## 2. Results

### 2.1. Variability: Elemental

The results from the two-way ANOVAs found no significant interactions and, for Mg, K, Fe and Zn, there were statistically significant changes in phloem exudate concentration with maturity for all four elements (*p* < 0.001 for all four elements). For the time of day groupings, only K and Fe were found to have statistically significant differences in phloem exudate concentration (*p* = 0.043 and <0.001, respectively).

**Figure 1:**
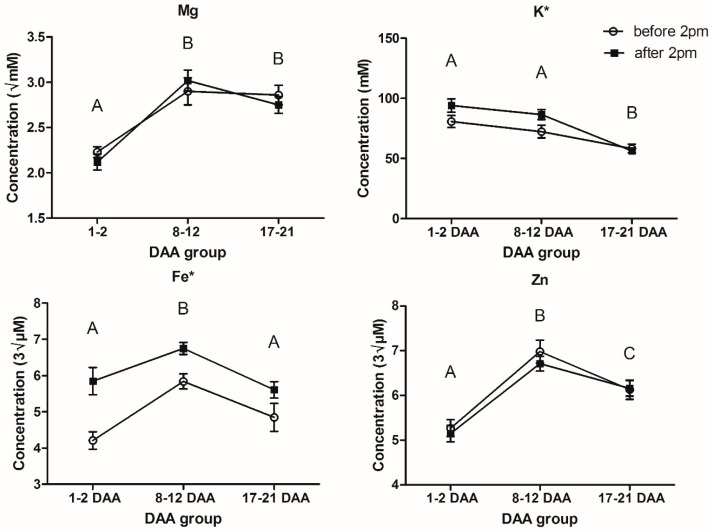
Mean concentrations ± standard error of Mg, K, Fe and Zn in the phloem exudate sampled before and after 14:00 for different maturities. Letters indicate significant differences between maturities at *p <* 0.05; * indicates significant differences between time of day at *p <* 0.05 (DAA, days after anthesis; sample sizes are listed in Table S1, while Table S3 presents the mean and SE for elemental concentrations at each sampling point).

The difference between time of day groupings for K and Fe saw an increase from the early sampling to the late sampling. On average across all maturities, K concentration increased by 8.52 ± 4.184 mM (Table 1) between phloem exudate sampled before 14:00 when compared to after 14:00, which is equivalent to a 1.1-fold increase. It can be seen in Figure 1 that this difference is mainly accounted for in the earliest maturities with nearly no difference observed at the end of grain loading (17–21 DAA). Fe was also found to increase in concentration from before 14:00 to after 14:00, with an average increase of 1.10 ± 0.252 $\sqrt[{3}]{\mu M}$ or a 1.2-fold increase. In Figure 1, the change in Fe phloem exudate concentration remains relatively consistent across all maturities.

For Mg, Fe and Zn, there was a significant (*p <* 0.001, Table 2 and Figure 1) increase in phloem exudate concentration from the start of anthesis (1–2 DAA) to the start of peak grain loading (8–12 DAA). From the start of peak grain loading (8–12 DAA) to the end of grain loading (17–21 DAA), we found that there was a significant decrease in phloem exudate concentration for K, Fe and Zn (*p <* 0.001 for K and Fe, *p* = 0.004 for Zn, Table 2 and Figure 1). It was also found that the Zn phloem exudate concentration at the end of grain loading was still significantly higher by 0.95 ± 0.222 cube root µM than the concentration at the start of anthesis (1–2 DAA), with a 1.2-fold difference (Table 2).

### 2.2. Within-Day Variability: Metabolite Profile

**Figure 2:**
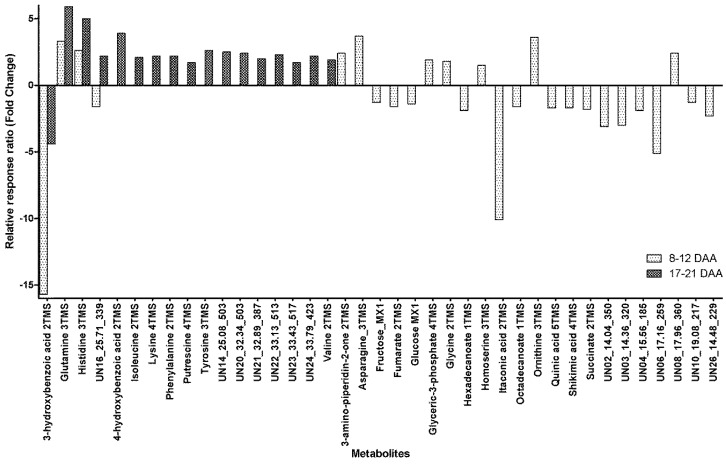
Fold change from before 14:00 to after 14:00 for metabolites in the phloem exudate metabolite profile with significant within-day variability in relative response ratios when collected at different maturities (8–12 DAA and/or 17–21 DAA) (DAA, days after anthesis; xTMS, Trimethylsilyl derivative where x is the number of TMS groups; yMX, methoxyamine derivatized product where y = 1 or 2; Table S4 presents the values for the relative response ratios).

Of the metabolites detected in phloem exudate, 39 metabolites were found to show significant within-day changes within the phloem exudate (before and after 14:00) at both maturity ranges sampled (8–12 DAA and 17–21 DAA). Four metabolites showed significant within-day variability at both maturity times sampled (Figure 2) with Glutamine and Histidine showing an increase of between 2.6- and 5.9-fold for samples taken before 14:00 to those taken after 14:00, whilst 3-hydroxybenzoic acid had a significant decrease of between 4.4- and 15.7-fold in the phloem exudate (Figure 2). The unidentified metabolite UN16 had a 1.6-fold decrease in the phloem exudate at 8–12 DAA but a 2.2-fold increase at 17–21 DAA from earlier to later in the day (Figure 2).

Apart from the above-mentioned metabolites, for samples taken at 8–12 DAA, there were seven metabolites with a significant within-day increase from early to late, ranging from 1.5-fold for Homoserine to 3.7-fold for Asparagine (Figure 2). At the same maturity time, there were 15 metabolites with a significant diurnal decrease between samples taken before and those after 14:00. The decrease ranged from 1.3-fold for Fructose to a 10.1-fold decrease for Itaconic acid (Figure 2). For samples taken at 17–21 DAA, there were 13 metabolites that all showed a significant increase from before 14:00 to after 14:00, with the increase ranging from a 1.7-fold increase for Putrescine to a 3.9-fold increase for 4-hydroxybenzoic acid (Figure 2).

**Figure 3:**
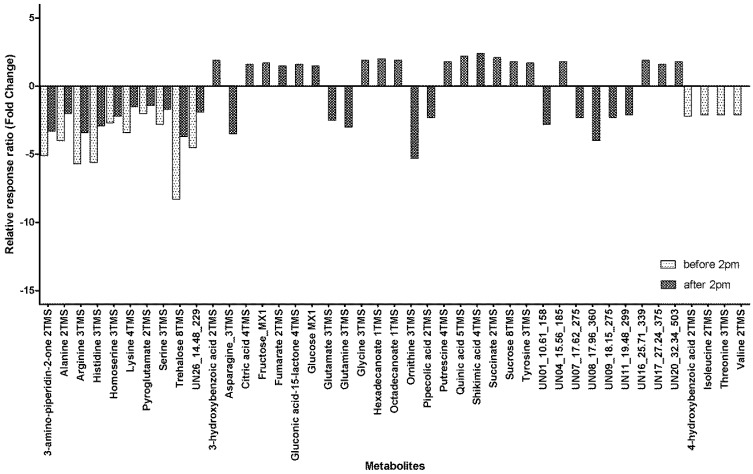
Fold change from 8–12 DAA to 17–21 DAA for metabolites in the phloem exudate metabolite profile with significant maturity variability in relative response ratios when collected at different times of day (before and/or after 14:00) (DAA, days after anthesis; xTMS, Trimethylsilyl derivative where x is the number of TMS groups; yMX, methoxyamine derivatized product where y = 1 or 2; Table S5 presents the values for the relative response ratios).

We have previously shown that metabolites vary with maturity [25] with 39 metabolites showing significant variability. In this work, we found a mostly similar relationship with 39 metabolites showing significant changes for samples taken after 14:00 but only 14 showing significant variability with maturity when sampled before 14:00. There were 10 metabolites that had significant maturity related variability when sampled at both time points (Figure 3). All 10 metabolites significantly decreased in the phloem exudate as the plant matured, with the decrease ranging from 1.4-fold for Pyroglutamate sampled after 14:00 to an 8.3-fold decrease for Trehalose sampled before 14:00. An additional four metabolites showed significant maturity related differences for samples collected before 14:00 and these metabolites all showed a decrease of around two-fold (2.1–2.2) in the phloem exudate with increasing maturity (Figure 3). For the remaining samples collected after 14:00, there were 10 metabolites that showed a significant decrease in the phloem exudate as the plants aged (Figure 3) and these ranged from a 2.1-fold decrease for UN11_19.48_299 to a 5.3-fold decrease for Ornithine. There were 19 metabolites that had a significant increase in the phloem exudate as the plants aged when sampled after 14:00; the increases ranged from a 1.5-fold increase for fumarate to a 2.4-fold increase for shikimic acid.

## 3. Discussion

In the work presented here, of the amino acids identified previously by Gattolin et al. [27], only leucine was not detected and aspartic acid was at the limit of detection for the analytical equipment used (data not shown). Of the remaining 16 amino acids, only proline was found to have no variability within the phloem exudate for either within-day or maturity change. Proline was also one of the amino acids reported to show within-day variability in the phloem exudate [27]. Of the other amino acids shown previously to have within-day variability, only arginine was found not to have variability in these conditions. It was however found to show a significant maturity difference with 3–5-fold decrease in the phloem exudate as the plants matured for both sampling points in the day (Figure 3). A possible explanation for the lack of within-day variability is that arginine and proline had the smallest reported regression coefficients [27]. This indicates that, even though change was significant, the within-day change observed was small. Unfortunately, the actual change was not reported. Gattolin et al. [27] also reported on a different genotype (Paragon) and on plants that were three weeks old, which may have led to the differences in diurnal fluctuations within the phloem exudate. This may also help to explain the significant diurnal variability observed for glutamine, glycine and ornithine (Figure 2), which were quantified but not found to have diurnal variability [27]. Of most importance, this work confirms that there is diurnal variability in amino acid composition within the phloem exudate, based on an increase in the amount of the amino acids present as the day progresses.

Links have been made between diurnal and circadian rhythms and the activity of Fe transport [18]. These include the production of compounds that enhance Fe uptake into the roots [8,9,11] and storage within the leaf [14]. In the work presented here, we provide evidence linking the observed role of circadian rhythms on Fe homeostasis to the transport of Fe within the phloem exudate. We observed on average a significant increase in Fe concentration in the phloem exudate of 1.10 ± 0.252 cube root µM (*p <* 0.001) from before 14:00 to after 14:00 across the grain loading period (Table 1). This 1.2-fold change in phloem exudate Fe concentration occurred within an approximately 3 h period (Table S1) and appears to be relatively consistent across maturities (Figure 1). This consistent relationship would link into previously mentioned relationships between circadian rhythms and Fe homeostasis [8,9,11,14]. With only two time points sampled in this study, further samplings at a greater range of day times could provide a closer link to the critical circadian pathways responsible for the observed difference of Fe phloem exudate concentration. In the early stages of this work, attempts were made to sample phloem exudate at an earlier time point, approximately 10:00 (data not shown), but we found that getting successful phloem exudate collections almost impossible due to low flow rates from severed stylets hampering collection efforts.

The only other element found to have significant variability at different times of day was K with a 1.1-fold increase from before 14:00 to after 14:00 (Table 1), and it appears that this relationship diminishes towards the end of grain loading, as can be seen in Figure 1, and also with the significant 1.5-fold decrease in K phloem exudate concentration from peak grain loading (8–12 DAA) to the end of grain loading (17–21 DAA) (Table 2). The main role of K in the phloem exudate is in maintaining turgor through its osmotic potential [28,29]. K has also been found to be involved in the process of loading both sucrose and amino acids into the phloem exudate [30,31] and the re-uptake of sucrose along the translocation stream [32]. A major role that K plays in assimilate transport is through its interaction with the proton symporter involved in assimilate loading [32]. Flow of K out of the phloem sap through potassium channels produces a negative potential within the phloem sap which enables the activation of the proton symporter, leading to loading of sucrose into the phloem sap [32]. This is likely to be of most importance for the scavenging of sucrose that has diffused from the phloem sieve elements, where the ATP required for activation of the proton pump may be in short supply [32]. The balancing of the osmotic potential is likely the main reason for a decrease in K concentration with maturity and may also explain the within-day changes observed in the phloem exudate K concentration as sucrose transport increases over the photoperiod is linked to the sink strength of the developing inflorescence. Unfortunately, we do not have metabolite results for the beginning of anthesis but it would be expected that assimilate concentration would increase over the course of the day and this can be observed with amino acids also showing an increase across the day, for the two maturities (Figure 2). This effect has been reported previously [27]. It is possible that the within-day difference is due to the loss of K from the phloem sap in an effort to scavenge sucrose lost due to diffusion. This may also explain why there is no diurnal difference observed at later maturities as the main role of K becomes one of sucrose recovery. Diurnal variation in phloem exudate K concentration may also be partially due to the role of the xylem in K transport from the roots and its supply to the shoots [28]. Reduced xylem flow due to lower transpiration at the leaf, early in the day may also reduce availability of K for phloem sap loading [28].

It is thought that Fe and Zn follow a similar route into cereals with both being complexed by phytosiderophores released into the soil for complexing metal ions and subsequent uptake into the roots [33]. For long distance transport, the route for Fe and Zn transport is not so clear. In a computer model based on reported phloem exudate composition, it was estimated that NA would complex 99.0% of Fe^2+^ but only 19.3% of Fe^3+^, whilst under the same conditions 54.4% of Zn^2+^ would be complexed [5]. For the remaining Fe ions, glutamate was the main ligand responsible for binding Fe (0.5% of Fe^2+^ and 69.9% of Fe^3+^) [5]. Citrate was also found to play a role in the complexing of Fe^3+^, with the model showing 9.2% of Fe being complexed by citrate and this amount was also shown to vary by up to 55.1% under different Ca and Mg conditions [5]. In the case of Zn, the S containing amino acid cysteine accounted for the remaining Zn complexing ligand (41.2%), though a small amount of Zn (2.8%) was modeled to be in a joint complex of cysteine and histidine. This model does not include DMA and since the publishing of this modeling research, Fe and Zn complexes in rice have been reported [6] and demonstrates that in the phloem exudate of rice, Zn was present as a complex with NA whilst Fe was complexed with DMA in the Fe^3+^ form. The GC-MS method used here was unable to measure cysteine or phytosiderophores but we have reported glutamate. There was no significant within-day variability for glutamate, but there was however a significant 2.5-fold decrease in glutamate in the phloem exudate towards the end of grain loading for samples collected after 14:00 (Figure 3). This may help in partially explaining the significant decrease in phloem exudate Fe concentration from 8–12 DAA to 17–21 DAA (Table 2).

It has been reported in rice and barley that there is diurnal regulation of phytosiderophore synthesis and secretion from root tissues [10,11,34,35]. Though there is a relationship between deficiency of both Fe and Zn and the production of phytosiderophores, the import of Zn into the roots under sufficient conditions may not be an active process [36] and may only hinge on the activity of metal transport proteins such as the ZIP family of metal transporters, for loading into the long distance transport stream [37]. This difference in root uptake may help explain the significant diurnal change observed for Fe concentration in the phloem exudate and a lack of significant within-day variability for Zn. Recent work has found that in rice grown under Fe deficiency, there is a diurnal related increase in the production of DMA in the roots [11]. This increase was found to peak at 3–5 h after the start of light conditions (11:00–13:00) [11], which falls just after the first collection point presented in this work. In this work, we did not find that methionine, a precursor of DMA and NA, had significant changes for either maturity or time of day. However in previous work we found a significant 1.3-fold increase in methionine with maturity [25]. It would be of interest to explore phloem exudate changes under nutrient deficiency to see if methionine and other precursors show greater fluctuations in the metabolite profile.

Of the 79 metabolites detected by GC-MS, there were 26 that were unable to be identified [25]. Of these 26 “unknown” metabolites, 14 had significant within-day variability (Figure 2) and 10 showed significant maturity variability (Figure 3) within the phloem exudate. At least one of these unknown metabolites may be of interest and requires further exploration. For UN8_17.96_360, or Unknown 8 with a retention time of 17.96 min and a unique ion at 360 *m*/*z*, there was a significant 2.4-fold increase from morning to afternoon at 8–12 DAA and a 4.0-fold decrease from 8–12 DAA to 17–21 DAA for samples collected after 14:00. This follows a similar within-day and maturity behavior to what we observed for Fe. Further examination of the mass spectra enabled a tentative identification of a glutathione precursor Cys-Gly (data not shown). Glutathione is a critical precursor of S rich compounds involved in heavy metal detoxification [38] and has been found in an increased concentrations in the phloem exudate of plants under heavy metal stress, such as Cd [39] and As [40]. It is also understood that glutathione is a critical component of the S assimilation pathway [41]. The S pathway is critical to Zn use as it has been estimated that 68% of Zn binding domains across the living world consist of at least one S containing cysteine [42]. Further exploration of Unknown 8 and the other unknown metabolites may give further insight into the metabolic pathways in within-day regulation of phloem sap transport.

The work presented here demonstrates the complex nature of the composition of phloem sap, with changes occurring in both the metabolite and nutrient phloem sap profile at different times of day and at different maturities. The significant changes observed for Fe and K concentrations and for the metabolites glutamine and histidine during the early parts of grain loading help improve our understanding of the processes involved in transport and the loading of essential micronutrients into the grain. Developments in methodology are in progress to add further metabolites such as sulfur containing amino acids and phytosiderophores which were not possible with the GC-MS method used here. This will enable further exploration of the metabolites that are proposed to complex Fe and Zn for long distance transport and also may be involved in the binding of these essential nutrients within the grain.

## 4. Materials and Methods

### 4.1. Plant Material

Wheat (*T. aestivum* L. breeding line, SAMNYT 16) seedlings were grown in a growth room in 70 × 100 mm pots filled with Debco™ Green Wizard potting mix. Growth room conditions were 13/11 h (6:00/19:00) light/dark at 20 °C/10 °C with a minimum of 400 µmol m^−2^ s^−1^ photosynthetic photon flux density at the leaf surface. Plants were planted in staggered groupings of 20 plants, at weekly intervals. Plants were transferred to a greenhouse where aphids were introduced and kept there for a maximum of 48 h. Light/dark cycle of the growth room approximately matched that of the greenhouse atmospheric cycle. Each phloem exudate sample was collected from a new plant.

### 4.2. Aphid Stylectomy

Aphid stylectomy procedures were adapted from Downing and Unwin [43] as detailed in [25]. In brief, apterous aphids of the species *Sitobion miscanthi* (Indian grain aphid) were used. Aphids were introduced to the plant immediately below the wheat head and on the peduncle of the main tiller a minimum of 12 h prior to stylectomy. Stylectomy was performed using high-frequency micro-cauterization.

Collections were grouped based on plant maturity and collection start time. Tables S1 and S2 show the mean maturity in days after anthesis (DAA) and collection start time for samples analyzed for micronutrients (Table S1) and metabolites (Table S2) for each grouping variable.

The cut off for the two sample collection start times was 14:00, the mean start times for the “before 14:00” group were 11:35:32 ± SE 0:07:57 for micronutrient analysis and 12:26:23 ± 0:10:22 for metabolite profiling samples (approximately 5.62 and 6.44 h after light start, respectively). The mean start times for the “after 14:00” group were 15:33:02 ± 0:03:13 for micronutrient analysis and 15:47:40 ± 0:07:18 for metabolite profiling samples (approximately 9.60 and 9.79 h after light start, respectively) (Tables S1 and S2).

### 4.3. Microscope Measurement of Nanoliter Phloem Exudate Volumes

Volumes of phloem exudate samples were measured as reported previously [44]. For samples analyzed by ICP-MS, phloem exudate volumes of the complete sample were measured under water saturated paraffin oil.

For samples analyzed by GC-MS, sample volumes were derived from an estimated flow rate calculated from photo sequences taken during sample collection adjusted for evaporation as detailed in Palmer et al. (2013) [44].

### 4.4. Sample Analysis

Elemental analysis of phloem exudate samples for Mg, K, Fe and Zn concentrations was performed by ICP-MS (7500cx; Agilent Technologies, Santa Clara, CA, USA), using a method previously reported [26]. GC-MS analysis of phloem exudate samples for metabolites was carried out at Metabolomics Australia using a method modified from [45] as detailed previously [25].

### 4.5. Statistical Analysis

Data were analyzed using SPSS^®^ Statistics software (IBM^®^, version 23). All data were checked to ensure they met the assumption of normal distribution according to the Shapiro–Wilk test for normality and transformed where necessary to conform to this assumption. Issues with non-homogenous data, as found using the Levene’s test, are noted in Table 1 and Table 2 as well as in tables included in Data S2. For the micronutrient data, a two-way ANOVA was conducted and the estimated marginal means were used to compare the time of day with a Bonferroni correction. The maturity differences were explored using a Hochberg GT2 post hoc comparison. For the metabolite data, independent *t* tests were used to compare the relative ion area between two maturities and between the two sampling times. When significant results were identified, the fold change was calculated using the ratio between the mean of the two groups in question.

## Figures and Tables

**Table 1:** Pairwise comparison of time of day groupings for each element, based on estimated marginal means from a two-way ANOVA using a Bonferroni adjustment for the comparison.

Element	Within-Day Group		Mean Difference (I-J)	Std. Error	Sig.	Fold Change (I → J)	95% Confidence Interval for Difference	
	I	J					Lower Bound	Upper Bound
Mg ($\sqrt{mM}$) ^#^	before 14:00	after 14:00	0.032	0.119	0.790	−1.01	−0.203	0.266
K (mM)	before 14:00	after 14:00	−8.52 *	4.184	0.043	1.12	−16.783	−0.255
Fe ($\sqrt[{3}]{\mu M}$)	before 14:00	after 14:00	−1.10 *	0.252	0.000	1.22	−1.60	−0.61
Zn ($\sqrt[{3}]{\mu M}$)	before 14:00	after 14:00	0.12	0.193	0.536	−1.02	−0.26	0.50

* *p <* 0.05, ^#^ Levene’s test *p <* 0.05.

**Table 2:** Hochberg GT2 post hoc test results for comparing maturity groupings after two-way ANOVA.

Element	DAA Group		Mean Difference (I-J)	Std. Error	Sig.	Fold Change (I → J)	95% Confidence Interval	
	I	J					Lower Bound	Upper Bound
Mg ($\sqrt{mM}$) ^#^	1–2 DAA	8–12 DAA	−0.84 *	0.141	0.000	1.39	−1.18	−0.51
		17–21 DAA	−0.63 *	0.136	0.000	1.29	−0.96	−0.30
	8–12 DAA	17–21 DAA	0.22	0.120	0.148	−1.08	−0.05	0.48
K (mM)	1–2 DAA	8–12 DAA	4.97	4.994	0.685	−1.06	−7.08	17.02
		17–21 DAA	31.94 *	4.763	0.000	−1.56	20.45	43.43
	8–12 DAA	17–21 DAA	26.97 *	3.940	0.000	−1.47	17.46	36.48
Fe ($\sqrt[{3}]{\mu M}$)	1–2 DAA	8–12 DAA	−1.39 *	0.287	0.000	1.27	−2.08	−0.69
		17–21 DAA	−0.14	0.293	0.947	1.03	−0.85	0.57
	8–12 DAA	17–21 DAA	1.24 *	0.246	0.000	−1.23	0.65	1.84
Zn ($\sqrt[{3}]{\mu M}$)	1–2 DAA	8–12 DAA	−1.57 *	0.229	0.000	1.30	−2.12	−1.01
		17–21 DAA	−0.95 *	0.222	0.000	1.18	−1.49	−0.41
	8–12 DAA	17–21 DAA	061 *	0.188	0.004	−1.10	0.16	1.07

* *p <* 0.05, ^#^ Levene’s test *p <* 0.05.

## Supplementary Materials

The following are available online at http://www.mdpi.com/2218-1989/8/3/53/s1, Data S1, Table S1: Sample numbers, mean and standard error of sample maturities (DAA) and sampling start times (hours:minutes:seconds) for sample groupings used for diurnal variability elemental comparisons, Table S2: Sample numbers, average ± standard errors of sample maturities (DAA) and sampling start times (hours:minutes:seconds) for sample groupings used for diurnal variability metabolite profile comparisons, Table S3: Average elemental concentrations (mg/L) found in phloem exudate samples for each maturity and time sampling combination. The standard deviation and sample sizes for each grouping are also included, Data S2, Table S4: Metabolites in the phloem exudate metabolite profile with significant time of day variability (“after 14:00” minus “before 14:00”) when collected at 8–12 DAA and 17–21 DAA, Table S5: Metabolites in the phloem exudate metabolite profile with significant maturity variability (17–21 DAA minus 8–12 DAA) when collected before and after 14:00.
