# Supplementary material for: Changes in the Elemental and Metabolite Profile of Wheat Phloem Sap during Grain Filling Indicate a Dynamic between Plant Maturity and Time of Day
Source: Metabolites. 2018 Sep 19;8(3):53. doi: 10.3390/metabo8030053 (PMC6160947; doi:10.3390/metabo8030053)
Supplement: Supplementary file 1 [file metabolites-08-00053-s001.zip › supplementary data 1.pdf]

Supplementary Material 1

Changes in the elemental and metabolite profile of wheat phloem sap during grain filling indicate a dynamic between plant maturity and time of day

Lachlan J. Palmer[1*] and James C. R. Stangoulis[1]

[1] School of Biological Sciences, Flinders University, Bedford Park, South Australia 5042, Australia

*** Correspondence:** Lachlan Palmer: Lachlan.palmer@flinders.edu.au

Table S1: Sample numbers, mean and standard error of sample maturities (DAA) and sampling start times (hours:minutes:seconds) for sample groupings used for within-day variability elemental comparisons.

Factor	Grouping	n	Mean	Std. Error
DAA	1-2 DAA	32	1.2	0.07
	8-12 DAA	61	9.9	0.11
	17-21 DAA	77	18.9	0.11
start time	before 2pm	50	11:35:32	0:07:57
	after 2pm	120	15:33:02	0:03:13

Table S2: Sample numbers, average ± standard errors of sample maturities (DAA) and sampling start times (hours:minutes:seconds) for sample groupings used for within-day variability metabolite profile comparisons.

Measurement	Grouping	n	Mean	Std. Error
DAA	8-12 DAA	28	9.7	0.18
	17-21 DAA	26	18.7	0.16
collection start	Before 2pm	18	12:26:23	0:10:22
	After 2pm	36	15:47:40	0:7:18

Table S3: Average elemental concentrations found in phloem exudate samples for each maturity and time sampling combination. Also included are standard deviation and sample sizes for each grouping.

Element (transformation)	Maturity Grouping	within-day Grouping	N	Mean	Standard Error of Mean	95.0% Lower CL for Mean	95.0% Upper CL for Mean
Mg ($\sqrt{\text{mM}}$)	1-2 DAA	before 2pm	11	2.23	0.06	2.10	2.36
		after 2pm	19	2.12	0.09	1.93	2.30
	8-12 DAA	before 2pm	10	2.90	0.15	2.56	3.23
		after 2pm	48	3.02	0.11	2.79	3.25
	17-21 DAA	before 2pm	25	2.86	0.11	2.64	3.08
		after 2pm	48	2.75	0.09	2.57	2.94
K (mM)	1-2 DAA	before 2pm	12	80.69	5.03	69.62	91.75
		after 2pm	19	94.09	5.50	82.54	105.65
	8-12 DAA	before 2pm	10	72.34	5.28	60.40	84.28
		after 2pm	46	86.45	4.13	78.14	94.76
	17-21 DAA	before 2pm	26	58.24	3.53	50.97	65.51
		after 2pm	49	56.28	2.77	50.71	61.86
Fe ($\sqrt[3]{\mu\text{M}}$)	1-2 DAA	before 2pm	11	4.21	0.24	3.67	4.75
		after 2pm	17	5.85	0.38	5.05	6.65
	8-12 DAA	before 2pm	9	5.84	0.21	5.36	6.32
		after 2pm	44	6.75	0.17	6.41	7.08
	17-21 DAA	before 2pm	16	4.85	0.39	4.03	5.67
		after 2pm	31	5.61	0.23	5.15	6.08
Zn ($\sqrt[3]{\mu\text{M}}$)	1-2 DAA	before 2pm	11	5.27	0.19	4.85	5.69
		after 2pm	19	5.15	0.19	4.76	5.54
	8-12 DAA	before 2pm	10	6.98	0.25	6.40	7.55
		after 2pm	42	6.71	0.17	6.37	7.05
	17-21 DAA	before 2pm	24	6.13	0.22	5.68	6.57
		after 2pm	37	6.16	0.18	5.80	6.52

*note that mean and 95% confidence limits (CL) can be un-transformed by squaring or cubing where appropriate to get units without transformation.
