# Supplementary material for: Changes in the Elemental and Metabolite Profile of Wheat Phloem Sap during Grain Filling Indicate a Dynamic between Plant Maturity and Time of Day
Source: Metabolites. 2018 Sep 19;8(3):53. doi: 10.3390/metabo8030053 (PMC6160947; doi:10.3390/metabo8030053)
Supplement: Supplementary file 1 [file metabolites-08-00053-s001.zip › supplementary data 2.pdf]

Supplementary Material 2

Changes in the elemental and metabolite profile of wheat phloem sap during grain filling indicate a dynamic between plant maturity and time of day

Lachlan J. Palmer[1*] and James C. R. Stangoulis[1]

[1] School of Biological Sciences, Flinders University, Bedford Park, South Australia 5042, Australia

*** Correspondence:** Lachlan Palmer: Lachlan.palmer@flinders.edu.au

Table S4: Metabolites in the phloem exudate metabolite profile with significant time of day variability (‘after 2pm’ minus ‘before 2pm’) when collected at 8-12 DAA and 17-21 DAA.

Metabolite	transformation	DAA group	Sig. (2-tailed)	Mean Difference	Std. Error Difference	Fold change
3-hydroxybenzoic acid 2TMS	CBRT	8-12 DAA	0.000 un	-0.3	0.044	-15.7
		17-21 DAA	0.000	-0.2	0.03	-4.4
Glutamine 3TMS	CBRT	8-12 DAA	0.007	0.4	0.13	3.3
		17-21 DAA	0.034	0.4	0.16	5.9
Histidine 3TMS	Ln	8-12 DAA	0.009	1	0.34	2.6
		17-21 DAA	0.019	1.6	0.64	5
UN16_25.71_339	None	8-12 DAA	0.035	-0.006	0.0029	-1.6
		17-21 DAA	0.037	0.01	0.0051	2.2
Asparagine_3TMS	Ln	8-12 DAA	0.022	1.3	0.53	3.7
Ornithine 3TMS	None	8-12 DAA	0.012 un	0.2	0.059	3.6
3-amino-piperidin-2-one 2TMS	SQRT	8-12 DAA	0.015	0.3	0.099	2.4
UN08_17.96_360	CBRT	8-12 DAA	0.036	0.1	0.065	2.4
Glyceric-3-phosphate 4TMS	SQRT	8-12 DAA	0.049	0.1	0.054	1.9
Glycine 2TMS	None	8-12 DAA	0.040	0.01	0.005	1.8
Homoserine 3TMS	None	8-12 DAA	0.025	0.05	0.023	1.5
Fructose_MX1	None	8-12 DAA	0.045	-0.1	0.05	-1.3
UN10_19.08_217	None	8-12 DAA	0.027	-0.4	0.15	-1.3
Glucose MX1	None	8-12 DAA	0.027	-0.4	0.16	-1.4
Octadecanoate 1TMS	SQRT	8-12 DAA	0.025	-0.2	0.068	-1.6
Fumarate 2TMS	None	8-12 DAA	0.040 un	-0.009	0.004	-1.6
Quinic acid 5TMS	SQRT	8-12 DAA	0.042	-0.2	0.072	-1.7
Shikimic acid 4TMS	SQRT	8-12 DAA	0.036	-0.1	0.044	-1.7
Succinate 2TMS	None	8-12 DAA	0.025 un	-0.03	0.012	-1.8
UN04_15.56_185	None	8-12 DAA	0.005	-0.1	0.041	-1.9
Hexadecanoate 1TMS	SQRT	8-12 DAA	0.001	-0.3	0.08	-1.9
UN26_14.48_229	Ln	8-12 DAA	0.006	-0.8	0.27	-2.3
UN03_14.36_320	Ln	8-12 DAA	0.000	-1.1	0.24	-3
UN02_14.04_350	SQRT	8-12 DAA	0.003 un	-0.1	0.039	-3.1
UN06_17.16_259	None	8-12 DAA	0.000 un	-0.008	0.0014	-5.1
Itaconic acid 2TMS	None	8-12 DAA	0.001 un	-0.01	0.0027	-10.1
4-hydroxybenzoic acid 2TMS	None	17-21 DAA	0.000 un	0.2	0.035	3.9
Tyrosine 3TMS	None	17-21 DAA	0.018	1.3	0.49	2.6
UN14_25.08_503	None	17-21 DAA	0.041	0.1	0.044	2.5
UN20_32.34_503	InvCBRT	17-21 DAA	0.004	-1.1	0.32	2.4
UN22_33.13_513	SQRT	17-21 DAA	0.038	0.03	0.015	2.3
UN24_33.79_423	None	17-21 DAA	0.003	0.04	0.012	2.2
Lysine 4TMS	None	17-21 DAA	0.032	0.7	0.29	2.2
Phenylalanine 2TMS	None	17-21 DAA	0.030	1.6	0.68	2.2
Isoleucine 2TMS	None	17-21 DAA	0.044	2.1	1	2.1
UN21_32.89_387	None	17-21 DAA	0.001	0.007	0.0017	2
Valine 2TMS	None	17-21 DAA	0.044	3	1.38	1.9
UN23_33.43_517	None	17-21 DAA	0.005	0.007	0.0023	1.7
Putrescine 4TMS	None	17-21 DAA	0.044	0.6	0.28	1.7

un = inhomogeneous sample variances as determined from Leverne’s test of equal variances, Ln = natural logarithm, InvCBRT = inverse cube root, SQRT = square root, CBRT = cube root. *x*TMS = Trimethylsilyl derivative where *x* = the number of TMS groups; *y*MX = methoxyamine derivatised product where *y* = 1 or 2

Table S5: Metabolites in the phloem exudate metabolite profile with significant maturity variability (17-21DAA minus 8-12 DAA) when collected before and after 2pm.

Metabolite	transformation	Collection time	Sig. (2-tailed)	Mean Difference	Std. Error Difference	fold change
3-amino-piperidin-2-one 2TMS	SQRT	Before 2pm	.027	-0.3	0.10	-5.1
		After 2pm	.001	-0.3	0.084	-3.3
Alanine 2TMS	SQRT	Before 2pm	.042	-0.4	0.16	-4.0
		After 2pm	.011[un]	-0.2	0.088	-2.0
Arginine 3TMS	Ln	Before 2pm	.006	-1.7	0.55	-5.7
		After 2pm	.000	-1.2	0.30	-3.4
Histidine 3TMS	Ln	Before 2pm	.011	-1.7	0.59	-5.6
		After 2pm	.009	-1.1	0.38	-2.9
Homoserine 3TMS	None	Before 2pm	.026	-0.07	0.029	-2.7
		After 2pm	.000	-0.09	0.019	-2.2
Lysine 4TMS	None	Before 2pm	.001	-1.3	0.31	-3.4
		After 2pm	.010	-0.7	0.24	-1.5
Pyroglutamate 2TMS	None	Before 2pm	.006	-8.2	2.55	-2.0
		After 2pm	.024	-3.7	1.58	-1.4
Serine 3TMS	None	Before 2pm	.012	-8.1	2.86	-2.8
		After 2pm	.001	-5.1	1.40	-1.7
Trehalose 8TMS	Ln	Before 2pm	.008[un]	-2.1	0.53	-8.3
		After 2pm	.001	-1.3	0.36	-3.7
UN26_14.48_229	Ln	Before 2pm	.035[un]	-1.5	0.54	-4.5
		After 2pm	.024	-0.6	0.27	-1.9
Isoleucine 2TMS	None	Before 2pm	.037	-2.0	0.89	-2.1
Threonine 3TMS	None	Before 2pm	.034	-1.0	0.44	-2.1
Valine 2TMS	None	Before 2pm	.031	-3.5	1.46	-2.1
4-hydroxybenzoic acid 2TMS	None	Before 2pm	.022[un]	-0.08	0.030	-2.2
Shikimic acid 4TMS	SQRT	After 2pm	.009[un]	0.2	0.064	2.4
Quinic acid 5TMS	SQRT	After 2pm	.037[un]	0.2	0.11	2.2
Succinate 2TMS	None	After 2pm	.024[un]	0.04	0.017	2.1
Hexadecanoate 1TMS	SQRT	After 2pm	.005[un]	0.3	0.097	2.0
Glycine 3TMS	None	After 2pm	.002[un]	0.1	0.037	1.9
Octadecanoate 1TMS	SQRT	After 2pm	.007[un]	0.2	0.084	1.9
3-hydroxybenzoic acid 2TMS	CBRT	After 2pm	.023	0.05	0.020	1.9
UN16_25.71_339	None	After 2pm	.004[un]	0.01	0.0030	1.9
Sucrose 8TMS	SQRT	After 2pm	.040[un]	0.3	0.13	1.8
UN20_32.34_503	InvCBRT	After 2pm	.031[un]	-0.6	0.28	1.8
Putrescine 4TMS	None	After 2pm	.001	0.7	0.19	1.8
UN04_15.56_185	None	After 2pm	.013[un]	0.1	0.040	1.8
Tyrosine 3TMS	None	After 2pm	.010[un]	0.8	0.30	1.7
Fructose_MX1	None	After 2pm	.007[un]	0.2	0.072	1.7
Citric acid 4TMS	None	After 2pm	.014	0.2	0.067	1.6
UN17_27.24_375	None	After 2pm	.009	0.01	0.0043	1.6
Gluconic acid-1,5-lactone 4TMS	None	After 2pm	.023	0.3	0.11	1.6
Glucose MX1	None	After 2pm	.026	0.5	0.20	1.5
Fumarate 2TMS	None	After 2pm	.027	0.007	0.0030	1.5
UN11_19.48_299	CBRT	After 2pm	.010	-0.08	0.031	-2.1
UN09_18.15_275	CBRT	After 2pm	.001	-0.1	0.026	-2.3
UN07_17.62_275	Ln	After 2pm	.001	-0.8	0.22	-2.3
Pipecolic acid 2TMS	Ln	After 2pm	.042	-0.8	0.40	-2.3
Glutamate 3TMS	None	After 2pm	.004	-1.1	0.35	-2.5
UN01_10.61_158	CBRT	After 2pm	.000	-0.09	0.017	-2.8
Glutamine 3TMS	CBRT	After 2pm	.004	-0.4	0.12	-3.0
Asparagine_3TMS	Ln	After 2pm	.015	-1.3	0.49	-3.5
UN08_17.96_360	CBRT	After 2pm	.000	-0.2	0.051	-4.0
Ornithine 3TMS	None	After 2pm	.005	-0.2	0.057	-5.3

un = inhomogeneous sample variances as determined from Leverne's test of equal variances,
Ln = natural logarithm, InvCBRT = inverse cube root, SQRT = square root, CBRT = cube root. *x*TMS = Trimethylsilyl derivative where *x* = the number of TMS groups; *y*MX = methoxyamine derivatised product where *y* = 1 or 2
